# The Effect of Gap Reduction on Fluid Dynamics and Mass Transfer in Membrane Narrow Channels Filled with Novel Spacers—A Detailed Computational Study

**DOI:** 10.3390/membranes13010020

**Published:** 2022-12-23

**Authors:** Panagiotis Saliakellis, Chrysafenia Koutsou, Anastasios Karabelas

**Affiliations:** Laboratory of Natural Resources and Renewable Energies, Chemical Process and Energy Resources Institute, Centre for Research and Technology-Hellas (CERTH), Thermi, 57001 Thessaloniki, Greece

**Keywords:** membrane, spacer-filled narrow channels, channel-gap reduction effects, novel net-type spacer geometry, detailed flow-field computer simulations, pressure drop and mass transfer generalized correlations

## Abstract

The effect of gap thickness reduction 2h (i.e., the reduction h at each membrane surface) is studied on the flow field and mass transfer, in membrane channels filled with novel spacers, under conditions representative of those prevailing in RO desalination modules. The patented novel net-type spacers are comprised of regularly-spaced spherical nodes (in contact with the membranes) and interconnecting cylindrical filaments at the midplane of the channel. Advanced 3D flow simulations, performed at “unit cell” level, show the strong effect of a modest gap reduction on the flow field. Analysis of the computational results leads to new insights regarding flow development as well as to useful correlations of key process parameters (i.e., for friction losses, mass-transfer rates, wall shear stresses) accounting for the effect of gap reduction. Contrary to conventional spacers, the novel spacer geometry, under conditions of usual/modest channel-gap reduction, exhibits no stagnant flow zones and relatively high shear stresses on both the membranes and the filaments, which leads to desirable mitigation of fouling and a reduction in concentration polarization. The developed correlations can be adapted to existing advanced simulators of RO module performance for improved process design and optimization.

## 1. Introduction

The presently dominant desalination and water treatment membrane technology of Reverse Osmosis (RO) and Nanofiltration (NF) involves a multitude of spiral-wound membrane (SWM) modules, which are arranged in series within pressure vessels [1]. Therefore, the design and optimization of an entire RO/NF plant should be based on the optimization of the design and performance of the individual SWM modules, which has been the subject of extensive research and development in recent decades (e.g., [2,3]), focused mainly on the feed-flow membrane channels, filled with net-type spacer sheets. The latter are the key structural components of such SWM modules, as they determine and create the detailed geometry of the membrane channels where the desalination process occurs [4]. Consequently, research and development on feed spacers primarily involves their detailed geometric characteristics, which critically affect the flow field and basic process parameters (i.e., pressure drop, concentration polarization). Moreover, these characteristics significantly impact the common operating problems of membrane fouling and scaling (e.g., [5,6]).

To address the complicated issues of flow and mass transfer in narrow spacer-filled channels, significant numerical, theoretical and experimental work has been performed in restricted, small spatial domains (e.g., [7,8]). A common approach taken in this work is the investigation of phenomena at the smallest possible spatial domain, i.e., the “unit cell”, formed by the regular net-type spacers. Such detailed studies aim to understand the effect of spacer characteristics on fluid flow and mass transfer (e.g., [8,9,10,11]), thus enabling optimization of the feed-spacer geometry (e.g., [12,13,14,15,16]). Significant investigations have also reported on membrane fouling, bio-fouling and scaling (e.g., [17,18,19,20,21]). In addition to improving basic understanding, useful outcomes of such investigations have been the correlations of key process parameters (such as pressure drop and mass-transfer coefficients) with the main variables (i.e., the prevailing pressures, velocities, fluid properties), which are applicable at “unit cell” scale (e.g., [8,22]).

As a next step, for development of a reliable simulator of SWM performance at steady state, a sound methodology pursued in the authors’ laboratory [23,24] involves the incorporation of the above-mentioned generalized correlations into a comprehensive modeling framework of flow and mass transfer throughout a membrane sheet/envelope and the entire SWM module (i.e., [17,23,25]). A further goal is to develop a dynamic model (and respective simulator) that accounts for evolving membrane fouling (i.e., [26]); such a dynamic simulator, as a general-purpose tool, would enable reliable projections on the performance of a pressure vessel and further, of a desalination plant.

In the aforementioned studies at “unit cell” level (e.g., [2,4,11]), the gap and detailed geometry of the spacer-filled membrane channel (at feed-side) is idealized; indeed, the channel gap is taken equal to the nominal thickness of the net-type spacer, considering that the spacer-net is in line- or point-contact with the bounding flat membranes. For the purpose of this study, this gap will be denoted as the “nominal gap” (H). However, in practice, the following two main effects cause the real membrane–channel gap to be smaller than the “nominal gap”, thus leading to significant modification of detailed channel geometry and of the feed-side flow field: (i) In fabricating SWM modules, normal stresses, also referred to as “interlayer pressure” [27], are applied on the spacer-filled membrane envelopes, during their winding, to obtain a compact and rigid module. These compressive stresses tend to cause local deformation of the membrane (usually indentations [28]) and possibly of the spacer, thereby effectively reducing the channel gap [29]. (ii) Channel-gap reduction commonly occurs during operation due to foulants depositing on the membranes, leading to the formation and evolution of fouling layers [30,31]; in the present study of feed-flow and mass-transfer characteristics, the flat surface of such layers will be considered. The total channel-gap reduction (due to the above effects), considered in this study, is defined as 2h; i.e., there is a thickness reduction h at each membrane surface, with a real channel gap (H—2h). For an improved understanding and comprehensive modeling of the desalination processes in SWM modules (outlined above), the effect of channel-gap reduction should be properly quantified. However, to the best of authors’ knowledge, such effects have been disregarded in the literature. Only recently have the authors of this paper investigated gap reduction effects in membrane channels filled with conventional net-type spacers comprised of two overlapping layers of parallel filaments [32].

Another related research and development activity is focused on the development of novel feed spacers (i.e., [33,34,35,36]) and their detailed geometrical features, which could lead to improved flow-field characteristics (e.g., minimization of concentration polarization, elimination of dead-flow zones) and a mitigation of membrane fouling and scaling. Such a novel spacer type [33], recently patented [37], involves regularly-spaced nodes interconnected with thin filaments (at the midplane between the membranes). Therefore, different contact points and regions exist within the spacer nodes with the bounding membranes, compared with those of conventional spacers.

The scope of this work is to study in detail the effect of gap reduction on flow field and mass transfer, in channels filled with the above-mentioned novel spacer, under conditions representative of those prevailing in RO desalination plants. For this purpose, 3D flow simulations were performed in “unit cells” by employing advanced numerical techniques described in previous publications [4,8,33]. The computational results are analyzed, leading to useful insights and correlations of key process parameters accounting for the effect of gap reduction.

## 2. Geometry and Parameters

**Figure 1:**
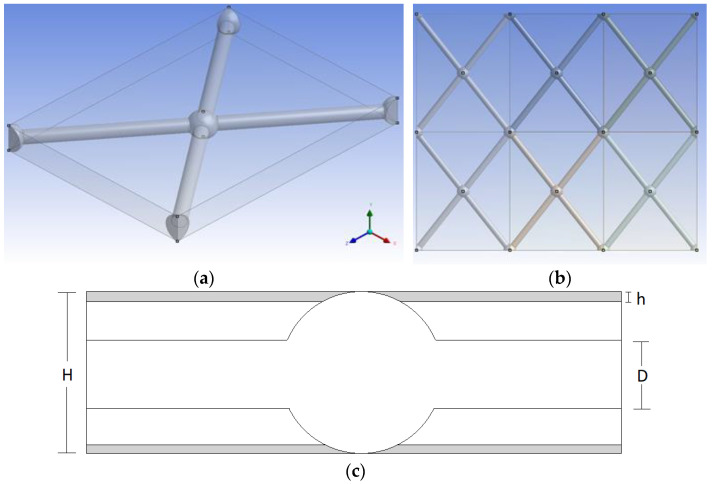
(**a**) The “unit cell” geometry for 3D simulations of the flow field and mass transfer. (**b**) Top view of the spacer pattern created by multiple unit cells; spacer between upper and lower membrane-bounding surfaces. (**c**) Cross-sectional view of membrane channel with spacer, indicating gap reduction h at each membrane surface, diameter of node (H) and of connecting filaments (D).

The flow domain considered in this study is formed by a novel, net-type spacer sheet in a narrow membrane channel, as described in previous work [16]. In brief, this spacer is comprised of spherical nodes of a diameter equal to the nominal channel gap H of the SWM envelopes, which are interconnected with cylindrical filaments of diameter D, equal to half the channel gap (D = H/2). Additional characteristics defining the spacer geometry are the distance ratio between parallel filaments over the filament diameter (L/D) and the angle between crossing filaments, β. Another parameter is the flow attack angle α, which is considered equal to 90^o^ (in respect of channel width/lateral direction z, Figure 1), to ensure that the two channel surfaces have symmetrical flow and mass-transfer characteristics, thus leading to similar concentration polarization and fouling behavior. For the cases studied here, a ratio of L/D = 12 and angle β = 105° was chosen (Figure 1). This particular spacer geometry has already been investigated [33] for the nominal channel gap H (i.e., h = 0), through detailed DNS computations; the respective results were validated with experimental measurements of pressure drop and mass transfer. Moreover, it was shown in a previous study [8] that these parameter values, for a conventional spacer, lead to a satisfactory or near-optimum flow field. In the present study, a thickness reduction h is considered to exist, uniformly on both flat membrane surfaces of the channel, due to uniform compression at both sides (e.g., [29,32]), or perhaps due to uniform deposition of foulants on both bounding membranes.

## 3. Numerical Simulations

As in previous studies (i.e., [4,8,32,33]), the fluid flow is considered to be periodic in respect to a unit cell. The unit cell for this study is comprised of neighboring spherical nodes and their connecting cylindrical filaments, as shown Figure 1a. A top view of the spacer pattern (created by multiple unit cells) is shown in Figure 1b. Therefore, for such flow-field and mass-transfer simulations, periodic boundary conditions are employed in conjunction with detailed computations at the unit cell level.

Assuming the fluid to be Newtonian and incompressible, momentum and mass transport are governed by the Navier–Stokes continuity and mass-transfer equations, respectively:

∂**u**/∂t + **u∙**∇**u** = −∇P + (1/Re) ∇^2^ **u** (1)

∇**u** = 0 (2)

∂C/∂t + **u∙**∇C = (1/Pe) ∇^2^ C (3)

The typical RO permeation velocities are much smaller in comparison to the cross-flow velocities, justifying the assumption of impermeable channel/membrane walls. Hence, no-slip and no-penetration boundary conditions are applied to the channel surfaces as well as to the filament surfaces. For the mass-transfer simulations, a uniform membrane wall concentration C_w_ is also assumed.

It is emphasized that the results of this study are obtained by performing a Direct Numerical Simulation (DNS) involving the aforementioned equations. Therefore, the whole range of spatial and temporal scales of developing turbulence is resolved, without introducing any approximation model for the flow structure. The simulations were carried out by using a commercial computational fluid dynamics (CFD) software package (ANSYS^®^ Fluent^®^) which employs the finite volume method (FVM). An adequate computational mesh is employed for both the spacer and the fluid region around it, ensuring a smooth solver convergence in all cases studied. The governing equations are integrated in time (for each iteration) by imposing a constant mean pressure gradient, until a statistically steady state is reached for the fluid flow and the concentration [4].

The following dimensionless Reynolds, Schmidt and Sherwood numbers are employed:

Re = D′ U/ν(4)

Sc = ν/D_c_ (5)

Sh = k D′/D_c_ (6)

where

1. D′ = D(1 − h’); (m)
2. D: Spacer filament diameter; (m)
3. 2h: Gap thickness reduction; (m)
4. h’ = h/D = 2h/H, Gap reduction parameter
5. H = 2D, Nominal channel gap (h = 0); (m)
6. G = (H − 2h) = 2D′, Gap thickness (m)
7. U: Mean superficial axial velocity in the channel of gap G = 2D′= (H − 2h); (m/s)
8. ν: Liquid kinematic viscosity; (m^2^/s)
9. D_c_: Species/salt diffusivity; (m^2^/s)
10. k: Mass-transfer coefficient; (m/s)

In the defined geometry, with channel width w and gap 2D′, the superficial velocity and Reynolds number are given as:

U = Q/(w∙2D′)(7)

Re = (D′∙U)/ν = Q/(2 w ν)(8)

Equation (8) shows that for constant flow rate Q, the Reynolds number remains constant for different gaps because the reduction in the channel gap (2D′) is compensated by an equal increase in the mean superficial velocity U. Consequently, the effects of pressure drop and other quantities can be interpreted and compared conveniently as a function of either Re number or flow rate Q, for different 2D′ gaps.

Typically, in RO modules, the axial superficial velocity varies in the range 0.1 to 0.4 m/s, whereas the manufacturers (e.g., [38]) recommend that the retentate pressure drop should not exceed 0.6 bar/m. Hence, the Reynolds number is usually in the range 50 to 200, based on the aforementioned range of superficial velocity and the spacer filament diameter D′ (Equation (8)). Additionally, the typical Schmidt number for brackish and sea water is roughly 600. In this study, the Schmidt number was varied in the range of 1 to 10^3^ to enable the development of generalized correlations of mass-transfer results. The main parameter values for the simulation cases of this study are summarized in Table 1; the investigated range of h/D values is realistic and selected on the basis of a previous relevant study [29].

## 4. Results and Discussion

### 4.1. General Flow-Field Characteristics

**Figure 2:**
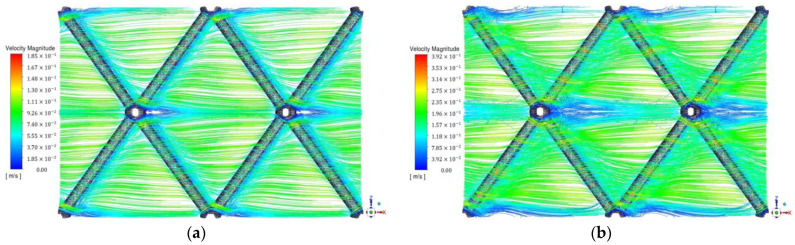
Detailed view of the fluid path lines for channel-gap reduction h/D = 0.10, at different Re numbers; (**a**) Re = 67, (**b**) Re = 149.

The effect of the channel gap on flow development is of particular importance, as it affects key membrane process parameters, including wall shear stress, mass transfer and concentration polarization phenomena. An analysis of such effects for both the reference/nominal-gap and the gap reduction (h > 0) for the conventional spacer has already been presented in previous works, in which the evolving flow field as well as the fluctuating wall velocities and shear stresses were considered [32]. The characteristics of the flow field and mass transfer for the novel spacer have also been presented [33] for the reference (h = 0) case. As previously shown, for the chosen spacer geometry of nominal gap H (h = 0), the flow is laminar at relatively small Re numbers (i.e., Re < 70). This also applies to the cases of various gap reduction h/D values, investigated here, where qualitatively the flow pattern tends to develop in the same manner. Indeed, as shown in Figure 2a, for (h/D) = 0.10 and Re = 69, the flow is laminar; however, at Re = 149 (Figure 2b) some flow unsteadiness is observed, which is also identified in the instantaneous velocity components.

The prevailing regular/symmetric flow pattern is depicted in the images of Figure 2, which show that the fluid streams tend to be aligned in the main flow direction (x-coordinate), passing through the narrow constrictions above and below the filaments. However, the flow appears to deviate in the downstream region of the cylindrical filaments, where a stream of vortices is created and moves attached along the filament walls. As observed in the base case (h = 0), these vortices tend to develop in a spiral manner downstream, along the cylindrical filaments, due to the three-dimensional nature of the flow and the component of a pressure gradient parallel to the filaments [3]. Notably, there are no closed recirculation (or “dead-flow”) zones, due to the highly symmetric spacer net in the channel midplane and the limited contact of the spacer with the two bounding membrane surfaces. The formation of a free vortex behind the spherical nodes of the unit cell is also noted, and is aligned with the main flow direction. This vortex is apparently reinforced from the interaction of the two above-mentioned moving vortices on both filaments, particularly at relatively high channel gap reduction values h/D. The images in Figure 2 indicate that these features, i.e., the vortices along the downstream side of cylindrical filaments and the vertical structures in the wake of the spherical nodes, are significantly enhanced with increasing Re, as expected. In following sections, additional clear, quantitative evidence is presented regarding the significant effect of channel-gap reduction on key flow-field parameters, such as the local shear stress distribution on the membranes and the filament surfaces.

### 4.2. Wall Shear Stress and Mass-Transfer Characteristics

**Figure 3:**
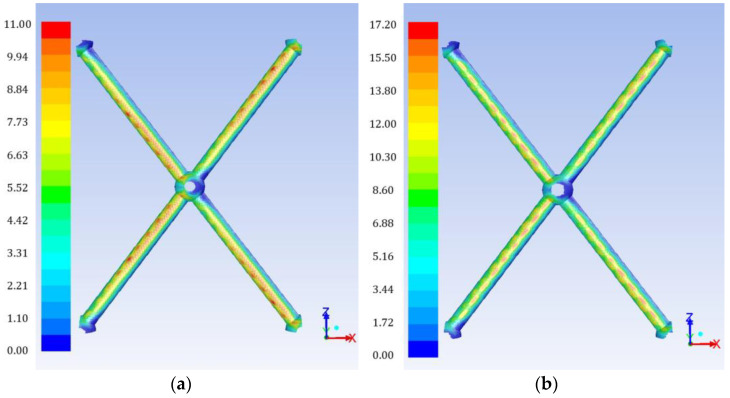
Instantaneous spatial distribution of the shear stress on the surface of the novel spacer filaments for two gap reduction values; (**a**) h/D = 0.075, (**b**) h/D = 0.10.

Typical flow characteristics related to the mass transfer and fouling phenomena are the distribution of shear stresses on the membrane surface and on the spacer filaments. In Figure 3, typical instantaneous shear stress distributions on the spacer cylindrical filaments are presented, for the two cases with the greatest gap reduction studied. As expected, high shear stress areas develop on the spacers, with the maximum values on the cylindrical-filament surface closest/opposite to the membrane surface, where the greatest flow constriction exists. A spatially-periodic pattern of those maximum shear stress values is evident in the particular instantaneous images of Figure 3. Therefore, a time-averaged spatial distribution would result in smooth and more uniform contours, as will be shown below.

**Figure 4:**
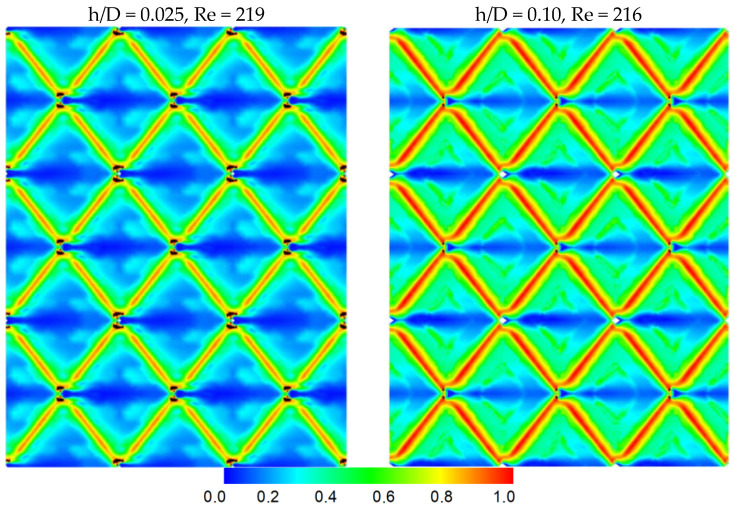
Typical spatial distribution of the local time-averaged shear stresses on the membrane surface for the same mean flow rate (and Re) and different gap reduction, i.e., h/D = 0.025 and 0.10.

The shear stresses on the membrane surfaces, obtained from the numerical simulations, are of great importance and directly related to the mass-transfer coefficients in the spacer-filled membrane channels. Figure 4 includes typical spatial distribution patterns of time-averaged shear stresses on the membrane surface, for two cases studied (i.e., h/D = 0.025 and 0.10), at the same Re number. It is evident that, even though the gap reduction h increases, the general shear stress distribution pattern remains symmetric and qualitatively quite similar (within the studied range of gap reduction); i.e., there are symmetric high-shear regions at the filaments, with significantly reduced shear stresses in the rest of the surfaces. It should be stressed, however, that near-zero shear stress values (associated with “dead-flow” zones) are essentially absent here, contrary to what is observed with conventional spacers [32] comprised of two-layer crossing filaments. As previously discussed, relatively low shear-stress values prevail in a restricted area right behind the spherical nodes, where the minimum flow velocities are also encountered.

It should be added that, as is generally well known for shear flow fields (including narrow-channel flow treated here, e.g., [8]), the spatial distribution pattern of the local mass-transfer coefficient (or Sh) is similar to that of shear stress (for the same Re), particularly for relatively high Sc numbers encountered in fluids treated by RO/NF membranes. Therefore, Figure 4 is also representative of the Sh spatial distribution.

**Figure 5:**
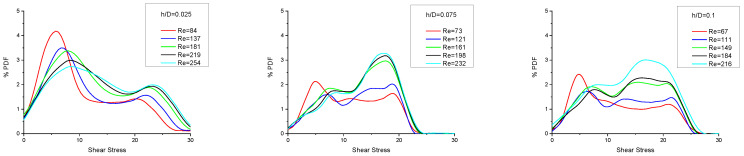
Percent probability density function of the dimensionless local time-averaged shear stresses on the membrane surface for the cases examined in this work. Shear stresses are normalized with the quantity μU/D.

In Figure 5, the comparison of probability density functions (PDF), for the studied three gap-reduction values h/D, suggests that, with increasing h/D, the dimensionless local time-averaged shear stresses tend to attain a somewhat more uniform distribution. This is attributed to the fact that as the effective gap (H–2h) is reduced, the velocities (as well as the wall shear stresses) near and underneath/above the cylindrical filaments tend to significantly increase and dominate. This is also reflected in the increasing mean pressure drop in the channel, as will be subsequently shown. Furthermore, a notable shift of the peak wall stress to higher values appears, particularly for the high gap-reduction values (cases 2 and 3), for Re > ~75, which is most likely due to the increased contributions of flow constrictions between filaments and the membrane surface. The beneficial effect of such shear stress distribution on the reduction in concentration polarization, mitigation of fouling and the overall improvement of membrane performance should be emphasized. Additionally, unlike the case of shear stress distributions with conventional spacers [32], which show a significant percentage of zero/near-zero values, no such trend is observed with the novel spacer geometry treated here.

**Figure 6:**
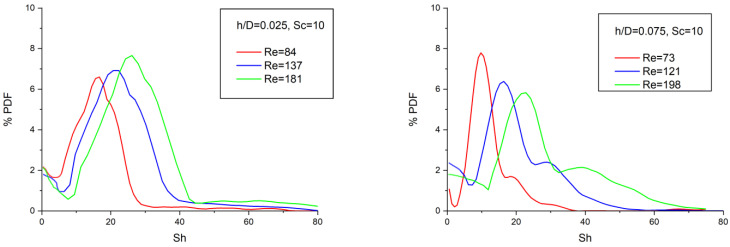
Probability density function of the local time-averaged Sh number at the membrane surface.

The mass-transfer simulations for the geometries corresponding to h/D between 0.025 and 0.10 were performed for the Schmidt number range of 1 to 1000. Figure 6 depicts the PDF distributions of the local time-averaged Sherwood number Sh for two indicatively selected cases of h/D, Sc = 10 and a typical range of Re number. These Sh number distributions tend to become broader with increasing Re number and extend to high values, signifying a reduced concentration polarization and likely, membrane fouling/scaling. Another notable feature in these distributions (as in the case of shear stresses) is the absence of zero and low Sh values (particularly at relatively high Re and gap reduction), which are associated with reduced concentration polarization and membrane fouling/scaling (e.g., [9,17,19,31]).

### 4.3. Friction Factor Correlations

**Figure 7:**
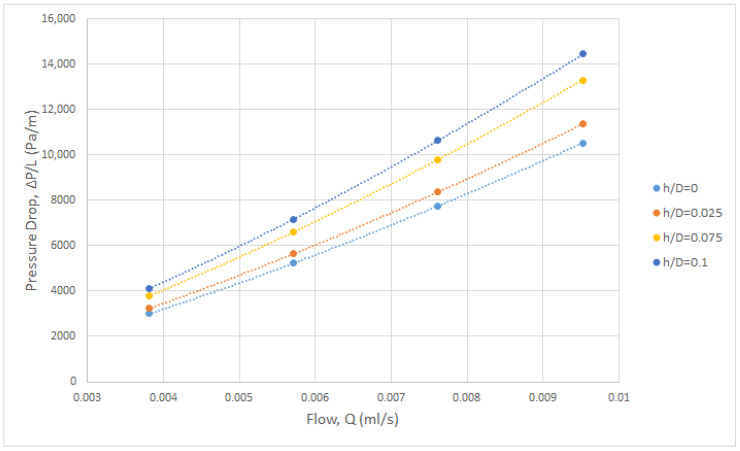
Computational results on the effect of gap reduction h on pressure drop ΔP/ΔL (Pa/m) in the retentate channel.

A significant issue investigated in this study is the effect of uniform channel-gap reduction h on the pressure drop in the retentate spacer-filled channels. For the reference case (h = 0), the pressure drop correlation for the same specific geometric spacer parameters (β = 105°, L/D = 12), is of the form f = 5.82 Re^−0.64^ as reported elsewhere [33]. This correlation will be integrated into the respective results for non-zero gap reduction (h/D) to obtain a unified correlation for all cases. The calculated pressure drop versus flow rate is plotted in Figure 7, for the parameter h/D range 2.5–10%, including the case of nominal gap h = 0. The employed flow rates Q correspond, for the unit cell computational domain, to superficial velocities in a range of practical interest, (i.e., ~0.10–0.25 m/s). As expected, the pressure drop increases with increasing gap reduction h. As the effective gap (H–2h) of the channel is reduced, the mean velocity increases due to the constriction of the free flow cross section, which in turn leads to increased pressure loss. Significant contributions to the latter are made by the wall shear stresses and the form drag due to flow around the cylindrical filaments and possibly the spherical nodes.

**Figure 8:**
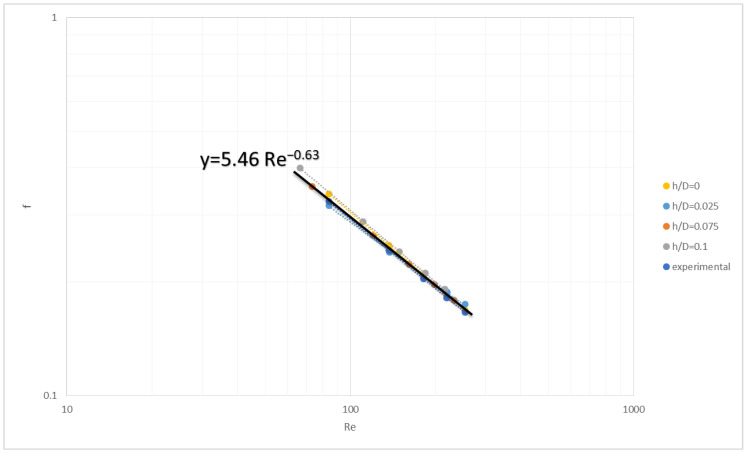
General friction factor versus Reynolds number correlation for various gap-reduction values h/D. Spacer parameters L/D = 12, β = 105°. Experimental data for case h/D = 0 are included.

To render the pressure drop data dimensionless, a friction factor f is defined as:

f = [dP/dL]/[(ρU^2^)/D′] (9)

where U is the superficial cross-flow velocity corresponding to the effective gap of the retentate channel (H–2h). Figure 8 depicts the results in dimensionless form, where it can be seen that for all the values of gap reduction h considered, the dependence of the friction factor f on Re number is very close to that of the reference/nominal gap (h = 0), including the experimental data (for h/D = 0) obtained in a previous study with the same spacer geometry [33].

Considering that both the pre-exponential factor and the exponent for each case are sensitive to even minor changes of the main parameters, it can be concluded that a satisfactory general correlation of the available data can be obtained. Therefore, the following general correlation, Equation (10) (depicted with a dark line in Figure 8) for the specific spacer geometry, is representative of all the aforementioned numerical data for gap reduction h/D, as well as the data from experiments [33] for (h/d) = 0.

f = 5.46 Re^−0.63^ (10)

The above correlation for the friction factor (as well as the following generalized correlation for mass transfer), accounting for gap reduction effects, has been developed for a particular set of spacer geometrical parameter (H, D, L) values, which are considered to be realistic. However, additional numerical simulation (and possibly experimental) work is required (of the type reported in this study) to select the optimum spacer geometrical parameter values for practical applications. An appropriate comprehensive methodology has been demonstrated by the present authors [29] to determine the optimum parameter values of spacers in the context of optimizing the overall spiral-wound membrane module design and performance. 

### 4.4. Mass-Transfer Correlations

**Figure 9:**
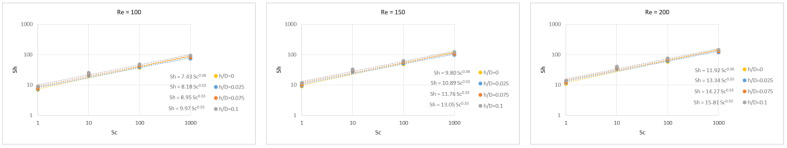
Theoretical predictions of Sh number dependence on Re number for various h/D values of the novel spacer and typical Re numbers.

As in the case of pressure drop, a key objective of the mass-transfer simulations is to establish a correlation of the Sh number accounting for gap reduction, including the Re and Sc dependencies. The computed space- and time-average Sherwood numbers are plotted in Figure 9, as a function of Sc number for constant Re numbers and h/D parameters studied in this work.

**Figure 10:**
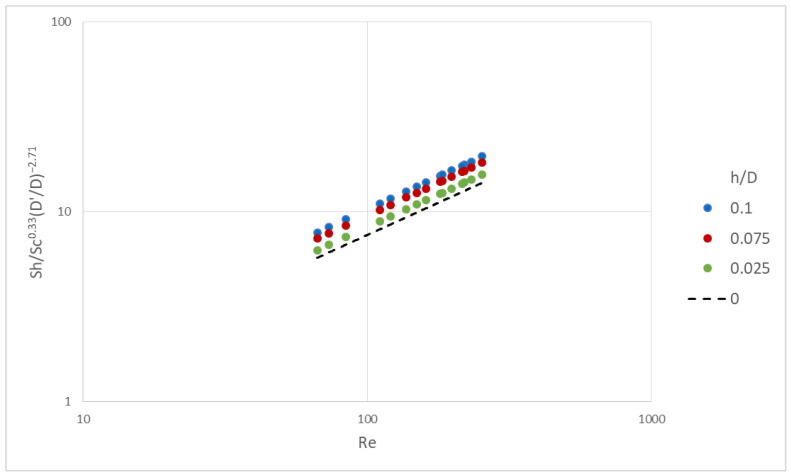
Generalized correlation of Sh as a function of Re number, accounting for D′/D. Solid line corresponds to Equation (11) and dotted line to Equation (12).

The case of the nominal channel gap (h/D = 0) is also included as a reference. The data sets for the three cases with h/D > 0 show that the mean Sh number depends on Sc number with a typical power-law exponent ~0.33. A final correlation can be obtained by plotting the dimensionless quantity [Sh/Sc^0.33^(D′/D)^−2.71^] versus Re number, as shown in Figure 10. More detailed information on how this dimensionless quantity was obtained can be found in the Supplementary Materials. These detailed data also show the expected trend of an increasing average mass-transfer coefficient and Sh, with increasing gap reduction, for a constant feed-flow rate.

The following generalized expression for mass-transfer rates (Equation (11)) satisfactorily correlates the data for the gap-reduction cases studied here (h/D = 0.025–0.10), i.e., within ±2% for the typical Re range of ~70 to 200.

Sh = 0.323 Re^0.69^ Sc^0.33^ (D′/D)^−2.71^ (11)

In comparison, the previously-reported [33] mass-transfer correlation (Equation (12)) for the nominal channel gap (h = 0) geometry, also depicted by a dotted line in Figure 10, deviates from Equation (11) by approx. −2% to −3%, for the same Re number range (~70 to 200).

Sh = 0.33 Re^0.68^ Sc^0.36^ (12)

## 5. Concluding Remarks

Results of numerical simulations are presented regarding the realistic case of gap reduction of the membrane channels, filled with a novel spacer, and the effect of such reduction on the flow-field and mass-transfer characteristics. The novel (net-type) spacer geometry consists of spherical nodes of diameter H = 2D, symmetrically placed (in a parallelogram pattern) and interconnected with cylindrical filaments of diameter D. A uniform channel-gap reduction h is considered at both membrane surfaces. In practice, such a gap reduction would occur due to compression (effected on membrane envelopes) during SWM module fabrication and/or due a uniformly-distributed fouling layer. The commonly employed “unit cell” approach is followed, with “cell” geometric parameters L/D = 12 and β = 105°. Direct numerical simulations (DNS) were performed for three cases of gap reduction parameters (i.e., h/D = 0.025, 0.075 and 0.10), and typical ranges of Reynolds and Schmidt numbers.

At relatively low Re values (<~70), the general flow field characteristics are those of steady laminar flow, regardless of the gap reduction, with low-velocity vortices moving along the (downstream side of) the spacer–filament surfaces. At greater Re numbers (>70), the flow becomes progressively unstable, with somewhat more intense vortices moving behind the cylindrical filaments. A vortical structure also appears to develop in the wake of each spherical node, particularly enhanced at increased gap-reduction values h/D. Unlike the case of conventional/commercial spacers (comprised of two overlapping layers of filaments), no stagnant flow zones are observed with the novel spacers in all studied cases.

In general, the channel gap significantly affects the key flow parameters, i.e., the channel pressure drop, shear stresses and mass transfer. As expected, these quantities tend to increase with increasing gap reduction. By employing the computational results, a generalized friction factor expression was developed for the novel spacer geometry, which satisfactorily correlates all the pressure drop data (for gap reduction, i.e., h/D > 0) as well as data from a previous numerical and experimental study for the nominal gap thickness (h/D = 0).

The mass-transfer characteristics of the studied spacer geometries are significantly influenced by the prevailing relatively high shear stresses, with maximum values on the membrane surface and on the cylindrical filament surfaces nearest to the membrane wall, i.e., where the greatest flow constrictions exist. Regarding the shear stresses on the membrane surface, it is shown that the pattern of local time-averaged shear stress distribution remains symmetric (similar to the spacer geometry) and unaltered with increasing gap reduction h. However, in the high-shear regions underneath and near the filaments, the shear stresses tend to increase significantly. The spatial distributions of Sh number follow the pattern of shear stresses, showing the expected increased values as Re increases, with the obvious beneficial effect of reduced concentration polarization at the membrane surface. Similarly, a beneficial effect of these flow characteristics is also expected in mitigating membrane fouling. Finally, as in the case of friction losses, a generalized mass-transfer correlation involving the dimensionless Sh, Re and Sc numbers was developed, taking into account the membrane effective channel-gap thickness.

The generalized pressure drop and mass-transfer correlations (developed herein for variable gap reduction) can be introduced and adapted to available (in this laboratory) appropriate software for a realistic simulation of SWM module performance under steady state as well as transient conditions, possibly induced by membrane fouling. However, considering that the present results were obtained with a specific set of spacer geometrical parameter values, there is a need to optimize these values in the context of optimizing the overall spiral-wound membrane module design and performance. To achieve this goal, additional modeling/simulation and experimental work is in the planning stage, including membrane fouling tests, where the present results will be employed. 

## Figures and Tables

**Table 1:** Conditions for performed simulation cases. Spacer geometry: L/D = 12, β = 105°.

Parameter	Case 1	Case 2	Case 3
Gap reduction parameter, h/D	0.025	0.075	0.10
Re number	84–255	73–233	66–216
Sc number	1–1000 (in all cases)		

## Data Availability

The data presented in this study are available on request from the corresponding author.

## Supplementary Materials

The following supporting information can be downloaded at: https://www.mdpi.com/article/10.3390/membranes13010020/s1: Figure S1: Time- and space-average Sh number on the membrane surface as a function of Sc number for various gap reduction parameter (h/D) values. Figure S2: Theoretical predictions of Sh number dependence on Re number for the novel spacer, for various gap-reduction h/D values. Figure S3: Theoretical predictions of Sh number dependence on Re number for various layer thicknesses of the novel spacer, for the same Sc number. Figure S4: Correlation of dimensionless Sh number parameter as a function of Re number, for gap reduction parameters h/D. Figure S5: Time- and space-average Sh number, on the membrane surface, as a function of ratio D/D′.
