# Supplementary material for: The Effect of Gap Reduction on Fluid Dynamics and Mass Transfer in Membrane Narrow Channels Filled with Novel Spacers—A Detailed Computational Study
Source: Membranes (Basel). 2022 Dec 23;13(1):20. doi: 10.3390/membranes13010020 (PMC9863175; doi:10.3390/membranes13010020)
Supplement: Supplementary file 1 [file membranes-13-00020-s001.zip › membranes-2064614-supplementary.pdf]

# Supplement

***The effect of gap reduction on fluid dynamics and mass transfer in membrane narrow channels filled with novel spacers - A detailed computational study***

*P. Saliakellis, C. P. Koutsou, A. J. Karabelas*
Chemical Process and Energy Resources Institute, Centre for Research and Technology-Hellas

## *A. Development of mass transfer correlations*

Supplementary information is provided herein regarding the development of generalized mass transfer correlations based on the data obtained from the numerical simulations. The satisfactory correlation with *~0.33* exponent is evident in the diagrams of **Figure S1**, where the *Sh* number is plotted as a function of *Sc* number for the range of channel gap numbers studied.

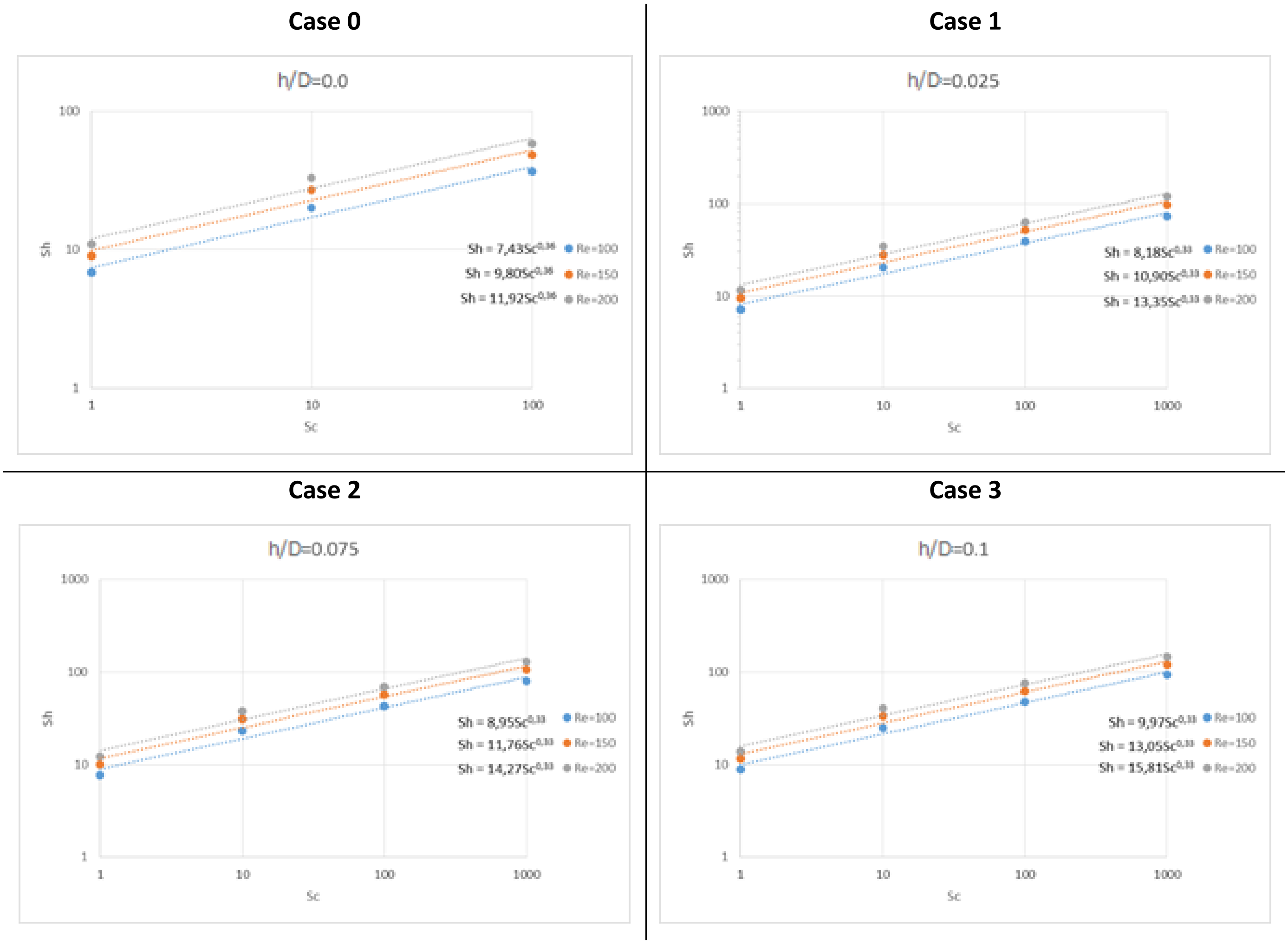


***Figure S1**: Time and space-average Sh number on the membrane surface as a function of Sc number for various gap reduction parameter (h/D) values.*

The *Sh* number results are also plotted as a function of *Re* number, as shown in **Figure S2**, for each *h/D* case studied, in the range of *Sc* numbers 1 to 1000. The case of nominal gap is presented for comparison, but for *Sc* number values only up to 100, as described in previous work [33]. While a general trend is followed in these correlations, the results for *Sc*=1000 exhibit a somewhat steeper slope, especially with increasing *Re* number. Alternatively, **Figure S3** shows data plotted for the *Sh* number as a function of *Re* number, for each *Sc* number studied, and the three *h/D* cases

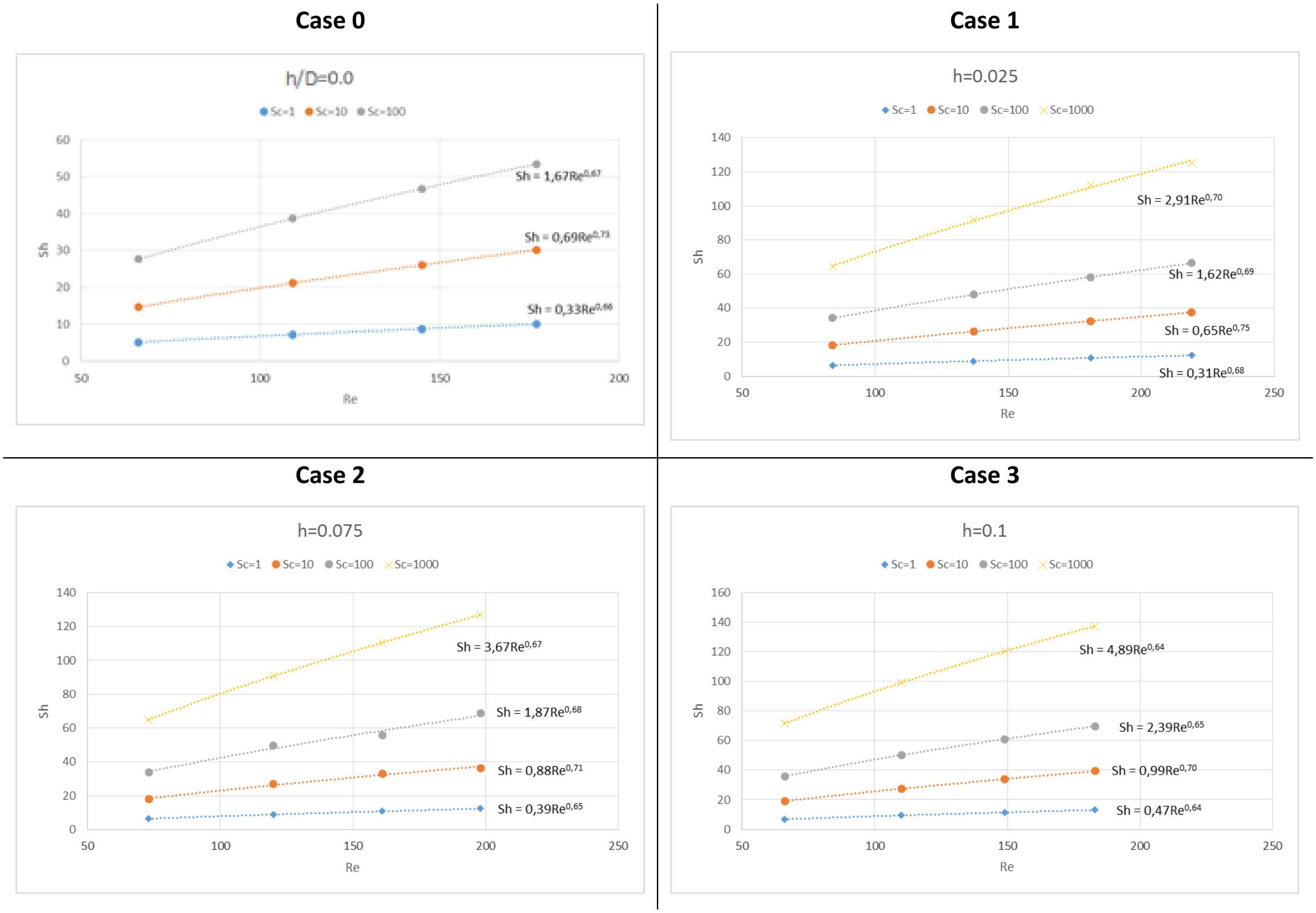


***Figure S2**: Theoretical predictions of Sh number dependence on Re number for the novel spacer, for various gap reduction h/D values.*

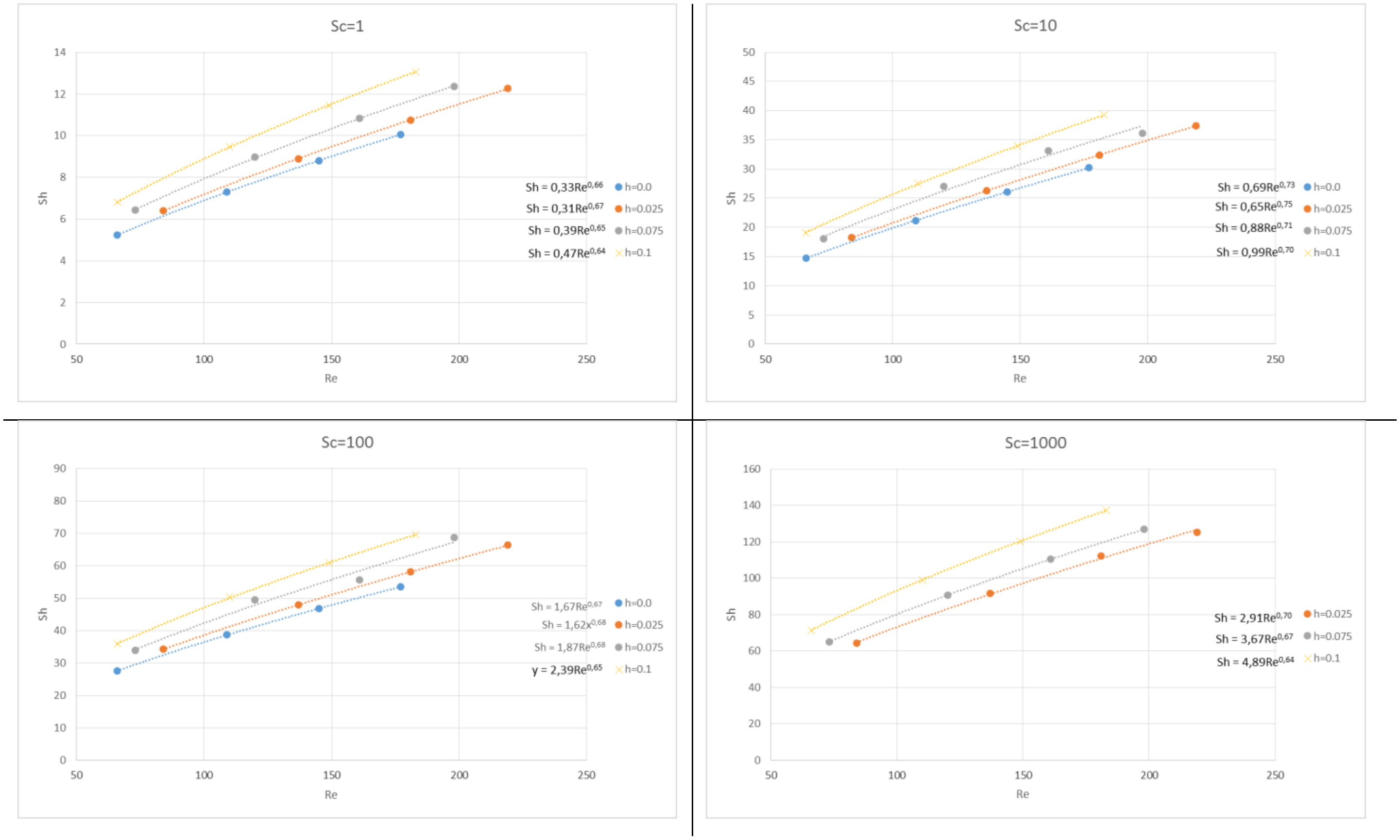


***Figure S3****: Theoretical predictions of Sh number dependence on Re number for various layer thicknesses of the novel spacer, for the same Sc number.*

In order to develop a generalized correlation, the data of **Figure S1** are replotted in **Figure S4**, in the form of $Sh/Sc^{0.33}$ as a function of *Re,* for each *h/D* value studied. Next, the *D'/D* dependence needs to be determined; thus, $Sh/Sc^{0.33}$ versus the ratio *D'/D* is plotted in **Figure S5**, showing a dependence approximated with an exponent *-2.71*.

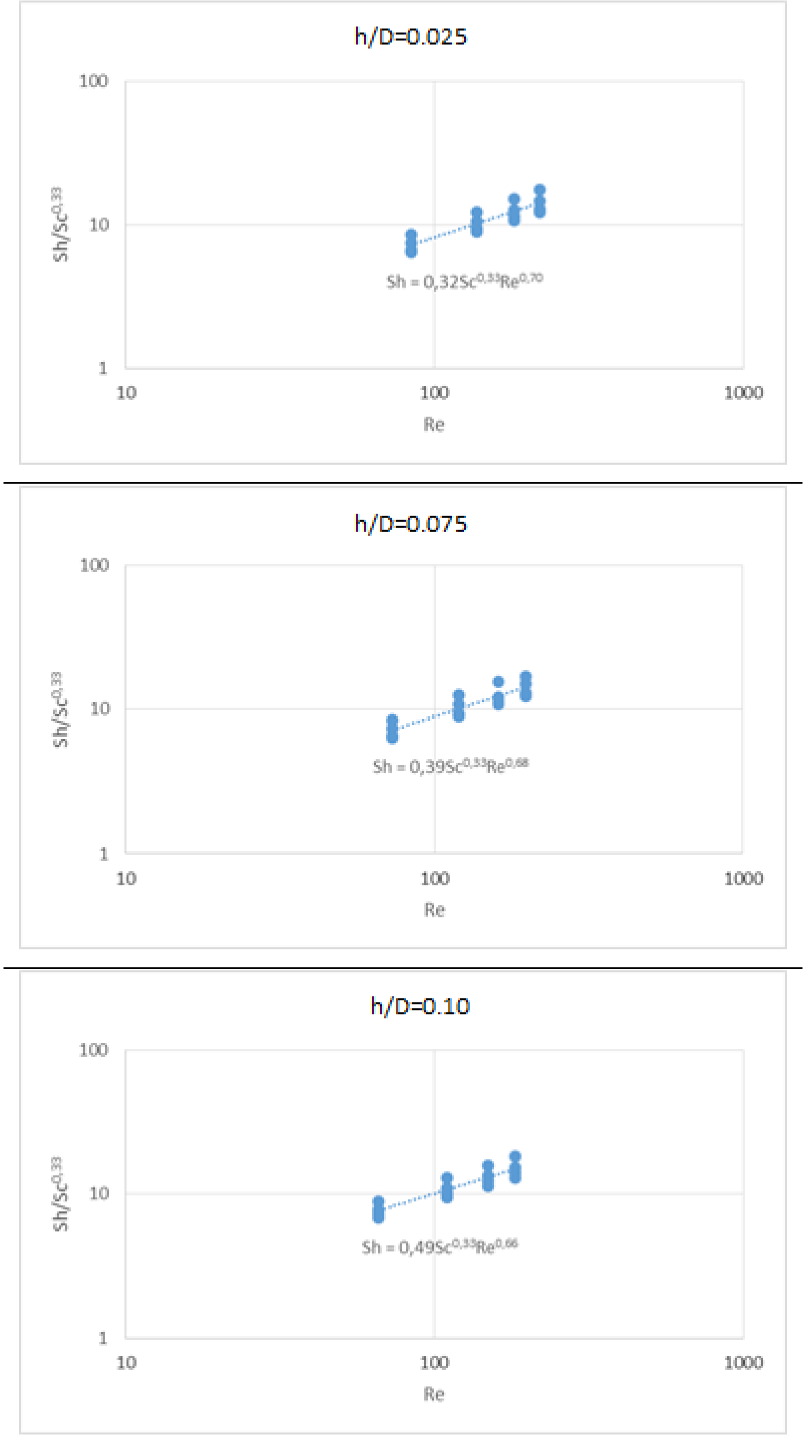


***Figure S4**: Correlation of dimensionless Sh number parameter as a function of Re number, for gap reduction parameters h/D.*

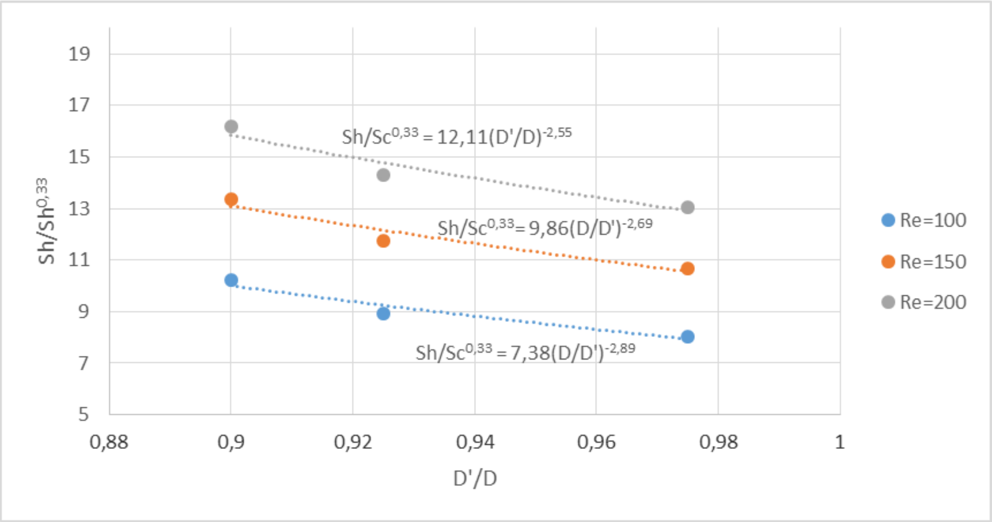


***Figure S5****: Time and space-average Sh number, on the membrane surface, as a function of ratio D/D'.*

### *B. Implementation of numerical computations*

- The simulations were executed in a computer with technical specifications: Intel® Core™ i7 CPU @ 3.07GHz and 12GB memory capacity.
- Computation time varied depending on input data (pressure drop and Sc number). Typical computation time was 2 days to reach/ensure a steady state (statistically), thus leading to time series with no or constant fluctuation.
- The computational mesh consisted of approximately $3x10^5$ elements (i.e. 61636 nodes with 328820 elements).
